# Supplementary material for: Design, Preparation and Properties of Polyurethane Dispersions via Prepolymer Method
Source: Molecules. 2023 Jan 7;28(2):625. doi: 10.3390/molecules28020625 (PMC9863305; doi:10.3390/molecules28020625)
Supplement: Supplementary file 1 [file molecules-28-00625-s001.zip › molecules-2120047-supplementary.pdf]

Supplementary Materials for

Study on the variables of emulsification and chain-extension process for polyurethane dispersions prepared by prepolymer method

Lijuan Sun，Hongmei Jiang*

Shanghai Huafeng New Material R&D Technology Co., Ltd. Pudong New Area, Shanghai, China

*Correspondence: jiang.hongmei@huafeng.com

Table S1 Sample designations of WPUR dispersions

Sample no.	Synthesis of prepolymer						Dispersion of prepolymer			
	PPG 2000 (mmol)	IPDI (mmol)	MPO (mmol)	DMPA（mmol）	TEA (mmol)	SDBS (%)	Water-adding step (One or two)	T of prepolymer（℃）	T of water（℃）	Ratio of EDA : H_2O
WPUR1	100	80	28.73	37.31	37.31	1.0	two	45	5	1:5
WPUR2	100	80	28.73	37.31	37.31	1.0	one	45	5	1:5
WPUR3	100	80	28.73	37.31	37.31	1.0	two	50	5	1:5
WPUR4	100	80	28.73	37.31	37.31	1.0	one	50	5	1:5
WPUR5	100	80	28.73	37.31	37.31	1.0	two	55	5	1:5
WPUR6	100	80	28.73	37.31	37.31	1.0	two	60	5	1:5
WPUR7	100	80	28.73	37.31	37.31	1.0	two	65	5	1:5
WPUR8	100	80	28.73	37.31	37.31	1.0	two	70	5	1:5
WPUR9	100	80	28.73	37.31	37.31	1.0	two	45	10	1:5
WPUR10	100	80	28.73	37.31	37.31	1.0	two	45	15	1:5
WPUR11	100	80	28.73	37.31	37.31	1.0	two	45	20	1:5
WPUR12	100	80	28.73	37.31	37.31	1.0	two	45	25	1:5
WPUR13	100	80	28.73	37.31	37.31	1.0	two	45	5	1:4
WPUR14	100	80	28.73	37.31	37.31	1.0	two	45	5	1:3
WPUR15	100	80	28.73	37.31	37.31	1.0	two	45	5	1:2
WPUR16	100	80	28.73	37.31	37.31	1.0	two	45	5	1:1

Table S2 Preparation and properties of WPUR dispersions with different adding emulsification water method.

Sample	One/two step	Solid content (wt%)	η (mPa·s)	Particle size/distribitution (nm)	CPH (mm)
WPUR1	two	40	180	170.6/0.157	1.2

WPUR2	one	40	50	294.9/0.565	16.7
WPUR3	two	40	160	164.8/0.114	0.7
WPUR4	one	40	40	278.4/0.470	9.6

Table S3 Preparation and properties of WPUR dispersions with different temperatures of prepolymer and emulsification water.

Sample	T of prepolymer （℃）	T of water （℃）	Solid content (wt%)	η (mPa·s)	Particle size/distribitution (nm)	CPH (mm)
WPUR1	45	5	50%	180	170.6/0.157	1.2
WPUR3	50	5	50%	160	164.8/0.114	0.7
WPUR5	55	5	50%	135	186.4/0.231	3.6
WPUR6	60	5	50%	142	193.1/0.315	5.2
WPUR7	65	5	failed	/	/	/
WPUR8	70	5	falied	/	/	/
WPUR9	45	10	50%	165	269.3/0.246	4.1
WPUR10	45	15	50%	159	240.1/0.362	4.7
WPUR11	45	20	falied	/	/	/
WPUR12	45	25	falied	/	/	/

Table S4 Preparation and properties of WPUR dispersions with different concentrations of the chain-extended amine.

Sample	Ratio of EDA : H2O	η (mPa·s)	Particle size/distribitution (nm)	CPH (mm)
WPUR1	1:5	180	170.6/0.157	1.2
WPUR13	1:4	165	180.2/0.184	1.4
WPUR14	1:3	132	200.3/0.216	2.5
WPUR15	1:2	124	237.5/0.311	7.9
WPUR16	1:1	96	239.7/0.359	8.3

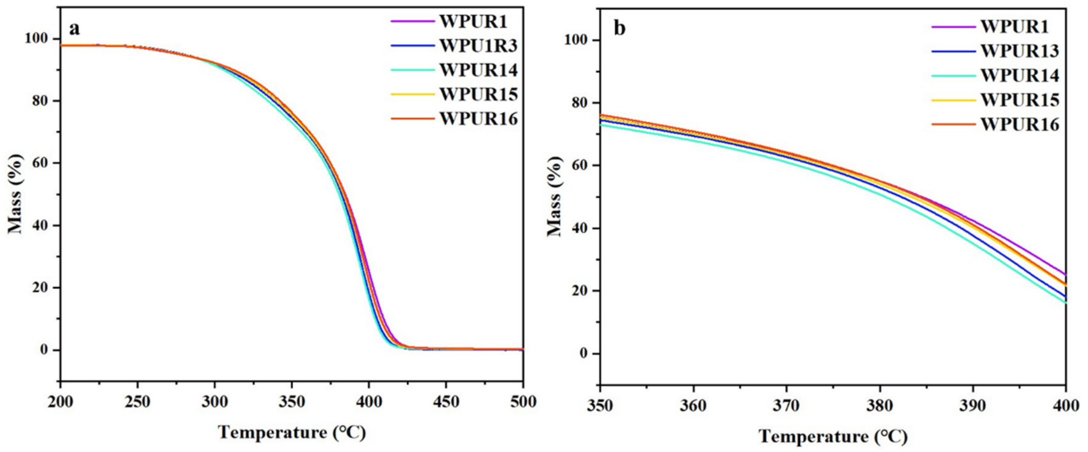

Figure S1 The TGA curve of WPUR films.
